# The Impact of Electronic Patient Portals on Patient Care: A Systematic Review of Controlled Trials

**DOI:** 10.2196/jmir.2238

**Published:** 2012-11-26

**Authors:** Elske Ammenwerth, Petra Schnell-Inderst, Alexander Hoerbst

**Affiliations:** ^1^Institute of Health InformaticsUMIT - University for Health Sciences, Medical Informatics and TechnologyHall in TyrolAustria; ^2^Institute of Public Health, Medical Decision Making and Health Technology AssessmentUMIT - University for Health Sciences, Medical Informatics and TechnologyHall in TyrolAustria; ^3^Oncotyrol Center for Personalized Cancer MedicineInnsbruckAustria; ^4^Research Division for eHealth and TelemedicineUMIT - University for Health Sciences, Medical Informatics and TechnologyHall in TyrolAustria

**Keywords:** Medical records, patient access to records, patient participation, patient portals, systematic review

## Abstract

**Background:**

Modern information technology is changing and provides new challenges to health care. The emergence of the Internet and the electronic health record (EHR) has brought new opportunities for patients to play a more active role in his/her care. Although in many countries patients have the right to access their clinical information, access to clinical records electronically is not common. Patient portals consist of provider-tethered applications that allow patients to electronically access health information that are documented and managed by a health care institution. Although patient portals are already being implemented, it is still unclear in which ways these technologies can influence patient care.

**Objective:**

To systematically review the available evidence on the impact of electronic patient portals on patient care.

**Methods:**

A systematic search was conducted using PubMed and other sources to identify controlled experimental or quasi-experimental studies on the impact of patient portals that were published between 1990 and 2011. A total of 1,306 references from all the publication hits were screened, and 13 papers were retrieved for full text analysis.

**Results:**

We identified 5 papers presenting 4 distinct studies. There were no statistically significant changes between intervention and control group in the 2 randomized controlled trials investigating the effect of patient portals on health outcomes. Significant changes in the patient portal group, compared to a control group, could be observed for the following parameters: quicker decrease in office visit rates and slower increase in telephone contacts; increase in number of messages sent; changes of the medication regimen; and better adherence to treatment.

**Conclusions:**

The number of available controlled studies with regard to patient portals is low. Even when patient portals are often discussed as a way to empower patients and improve quality of care, there is insufficient evidence to support this assumption.

## Introduction

### Background

The progress of modern information technology (IT) is changing and challenging health care. Clinical information systems as well as electronic health records have offered new opportunities for efficient and high-quality patient care [1].

The emergence of the Internet and of the electronic health record (EHR) has brought new opportunities for a new and more active role of the patient [2-4]. The patient’s role is changing from a patronized patient to an informed patient and further to a responsible, autonomous and competent partner in his or her own care [5]. An active integration of the patient in his/her treatment bears multiple potential benefits such as fostering the quality of care as well as the compliance of the patient [6,7].

One precondition for a more active patient’s role is to give the patient access to a providers’ documentation on previous or planned treatment. In many countries, patients have the right to access their clinical information whenever they request [8]. The Institute of Medicine argues that “patients should have unfettered access to their own medical information” and that this may help to increase quality of care and reduce medical errors [9]. However, patients demanding record access is not a common situation, due to cultural and practical reasons [10,11] and in part due to concerns by health care practitioners [8, 12]. Compared to paper-based solutions, information technological concepts such as electronic patient portals and personal health records (PHRs) seem to provide the opportunity to facilitate patients’ access to their clinical information.

PHRs have been defined as “a set of computer-based tools that allow people to access and coordinate their lifelong health information and make appropriate parts of it available to those who need it” [13]. PHRs focus on functionalities where patients can document health-related data and can, if wanted, make them available to others, for example to their health care providers or families [14]. PHRs are typically owned and administered by the patients themselves.

### Electronic Patient Portals

Electronic patient portals comprise provider-tethered applications that allow patients to electronically access health information that is documented and managed by a health care institution [15]. Patient portals are owned and administered by health care institutions (such as hospitals). As part of a patient portal, institutions may offer patients (typically web-based) access to selected clinical data that is governed by the respective institutions as part of the patients’ EHR. The patient can then access clinical data, read and print it, or integrate it into any (electronic or paper-based) type of patient-owned record. Besides providing access to EHR data, electronic patient portals may also offer additional services and functions to the patients. These include medication refills, appointment scheduling, access to general medical information such as guidelines, or secure messaging between a patient and an institution [15].

In order to provide a distinctive definition for the current review, we define electronic patient portals as the class of applications provided and maintained by health care institutions that primarily allow access to clinical EHR data and secondarily may offer functions and services that are targeted towards enhancing medical treatment. For reasons of simplicity, all of these applications are called patient portals, regardless of their actual implementation e.g. as part of a PHR.

### The Impact of Electronic Patient Portals

The idea of providing patients access to clinical information is not new. Traditionally, this has been done by providing a patient with paper-based copies of extracts from clinical documents [16-18]. Ross et al [8] have reviewed the effects of paper-based access to medical records and found that it has the potential for modest benefits for patient care, for example in enhancing doctor-patient communication, with only minimal risks such as increasing patient worry or confusion.

In 2007, Ferreira et al [10] reviewed 14 papers that dealt with the effects of electronic patient portals on medical practice. He concluded that some studies indicated benefits of electronic patient portals, for example by enhancing communication, but that the studies also showed patients’ concerns about confidentiality and understandability of the content. The majority of the papers reviewed were surveys and interviews with patients and clinicians, without including a control group. Therefore, these studies were not able to objectively identify any benefits of patient portals when added to traditional patient care.

To our knowledge, no systematic review of controlled studies on the impact of electronic patient portals has been conducted to date. With the further emergence of electronic patient portals in recent years, and the growing interest in evidence-based health informatics [19], we see the need to review the benefits of patient portals. The focus of this review is on the benefit of electronic patient portals for the patient, thus we focus on the impact of electronic patient portals on patient care. We did not predefine specific endpoints, but included all studies with endpoints, which were supposed to represent an impact on patient care by the study authors. For example, electronic patient portals may improve communication between provider and patient, or increase a patient’s adherence to medication treatment. Both could contribute to better patient care. Whether these benefits in fact arise when electronic patient portals are introduced needs to be shown by controlled trials and systematic reviews.

### Objectives

The main objective of this paper is to systematically review the impact of electronic patient portals on patient care by analyzing controlled studies on the use of patient portals. The structure of this paper and the presentation of the results follow the PRISMA statement for reporting systematic reviews [20].

## Methods

### Eligibility Criteria

Papers were eligible if they presented controlled studies on the impact of patient portals. Impact could be visible in outcome-oriented parameters such as changes in mortality or morbidity or in costs of care. Also process parameters such as changes in therapy adherence or in patient satisfaction with the provided care were included, even when these parameters are merely surrogates for clinical outcome. With regard to study design, we included experimental (e.g. RCT) or quasi-experimental (e.g. controlled before-after trials) studies.

In accordance with the definition given in the introduction for patient portals, patient portals are characterized by the following attributes:

- electronic applications, typically web-based
- provided and maintained by health care institutions
- targeted towards providing functionality to all or a subgroup of patients
- basic functionalities to access (a subset of) a patient’s clinical data
- optional, additional functionalities such as communication modules, prescription refills, appointment scheduling, or educational guidelines

The basic criterion for inclusion of a study was, however, that the application allowed the patient to access clinical data provided by a health care institution. We did not include papers that focused on telemonitoring systems, where the focus was on patients actively or passively providing data for their clinicians, or those that focused only on tailored messaging (e.g. of prevention information) from clinicians to patients such as Lin et al [21]. On the other hand, we included applications that were introduced as PHRs and looked for the functionality of an electronic patient portal, namely the possibility to access clinical information from a patient’s health care provider.

We did not limit the search to a specific clinical setting, thus we included portals both in inpatient and outpatient areas. We included studies independent of the patient subgroup or disease (e.g. general portals, but also portals for diabetes patients).

We limited the search to papers after 1990, as we did not expect to identify patient portal papers before that date. We excluded all papers where the intervention consisted of a paper-based copy of the medical record, as a systematic review on this topic has already been done [8]. We included papers in English, German and French.

### Information Sources

We performed a literature search in April 2012, in PubMed Cochrane Library, CINAHL, EMBASE, ACM Digital Library and UMIT’s own Evaluation Database for relevant studies. Bibliographies of the included studies as well as of the reviews of Ross et al [8] and Ferreira et al [10] were used to identify additional studies.

### Search

We used a combination of two queries (see Textbox 1). The first query searched for all papers about medical record systems that dealt with access to information or active patient participation. As the usage of MeSH headings was not consistent in all cases, we added a second query that looked at the term “patient portal” anywhere in the title or abstract.

**Description of Queries.**

*Query 1*:

("Medical Records Systems, Computerized"[mh] OR "Health Records, Personal" [mh] OR "Electronic Health Records" [mh] OR "Medical records" [mh])

AND

("Access to Information"[mh] OR "Patient participation" [mh] OR "Patient access to records" [mh])

AND

("1990"[PDAT] : "2011"[PDAT])

*Query 2*:

"patient portal" OR "patient web portal"

AND

("1990"[PDAT] : "2011"[PDAT])

After retrieving the results of the two queries, we imported all references to the Endnote reference manager and eliminated duplicates.

### Study Selection

Two authors independently screened the titles and abstracts of all references to confirm whether the inclusion criteria were fulfilled. Differences were resolved by having a third author judge the paper. For all preselected papers, full text versions were retrieved and two authors independently determined whether the inclusion criteria were fulfilled. Differences were again resolved by consulting a third author.

### Data Collection Process

Each included study was systematically described addressing clinical setting, type of intervention, type of study as well as outcome measures. Data extraction was done independently by two researchers. Results were compared and any differences were resolved by discussion.

### Data Items

The following data items were documented for each study:

- Time of study
- Clinical setting
- Type of patients included
- Description of intervention
- Description of control
- Study design
- Number of participants
- Outcome measures
- Study findings

### Assessment of Study Quality

The study quality was assessed using the methodology checklists for RCT studies and for cohort studies of the National Institute for Health and Clinical Excellence (NICE) [22]. These checklists were applied independently by two reviewers (PSI, EA). Differences in judgment were solved by discussion.

### Synthesis of Results

We systematically described the characteristics and results of the included studies in evidence tables. Further synthesis of results in the form of a meta-analysis was initially planned, but not possible due to the different outcome measures examined in the studies.

Results were ordered according to the type of outcome measured, namely objective criteria and subjective criteria.

## Results

### Study Selection

The queries in PubMed were performed in April 2012. Query 1 found 1,098 references, Query 2 found 52 references. Overall, when eliminating duplicates, we identified 1,136 references. We then checked Cochrane Library, CINAHL, EMBASE, ACM Digital Library and our own Evaluation Database at UMIT for relevant studies. We could not identify any further studies meeting our inclusion criteria. We also checked the reviews of Ross et al [8] and Ferreira et al [10] and citations in the included studies, but did not identify further studies.

From the identified 1,136 papers, only 13 had an experimental or quasi-experimental study design (Multimedia Appendix 1). Of those 13 papers, 5 papers [23-27] presenting 5 distinct studies were finally eligible and then analyzed in detail. Two papers [26,27] describe the same study. Therefore, an overall total of 4 controlled studies were included in the review.

### Study Characteristics

The 5 study papers presented evaluations of 4 different patient portals. Two papers [26,27] described different aspects of the same study. One portal was designed for patients undergoing IVF (in-vitro fertilization) treatment [23], one for diabetes mellitus patients [25], one for patients with congestive heart failure [26,27] and one was a general patient portal [24]. Three of the portals were located in the U.S. and one in the Netherlands. For details, see Table 1.

Three of the studies were randomized controlled trials (RCT); one was a retrospective matched cohort study. The number of participants in each study ranged from 81 to 6,402 patients. The studies evaluated the impact on a variety of outcome criteria. One study focused on changes in clinical outcome parameters, including HbA1c, blood pressure, LDL, and medication adjustments [25]. One study focused on changes of resource consumption, including office visit rates and telephone contacts [24]. One study focused on changes of more subjective parameters such as patient satisfaction, patient knowledge, and patient anxiety [23]; these were measured by validated questionnaires. The fourth study combined changes of mortality, treatment adherence and resource consumption (message number) with subjective parameters (subjective health status, patient empowerment, medication adherence) [26,27].

### Impact of Patient Portals on Outcomes

There were no statistically significant changes between intervention and control group in the 2 randomized controlled trials [25-27] investigating the effect of patient portals on endpoints measuring health or proxies for health (mortality, emergency room visits, hospitalizations, heart failure practice visits or risk factors). The use of patient portals showed no effect on all measurement scales to operationalize patient empowerment in one study [23].

Statistically significant changes in the patient portal group, compared to a control group, could only be observed for the following parameters: quicker decrease in office visit rates and slower increase in the number of telephone contacts [24]; increase in number of messages sent [27]; changes of the medication regimen [24]; and better adherence to treatment [27]. For details on study design and measured outcomes, see Table 2.

**Table 1:** Details of included studies – description of setting, intervention and control.

Author/ Year	Time of study (MM/YY)	Clinical setting	Inclusion criteria	Intervention group	Control group
Tuil, 2007 [23]	Start: 01/04 End: Unclear	Radbound University Nijmegen Medical Center, Fertility clinic	All adult couples that were scheduled for their first IVF (in-vitro fertilization) or ICSI (intracytoplasmic sperm injection) treatment cycle and had internet access during the inclusion period (Jan. – July 2004) were invited to participate	Access to a web site that offered: Access to general information about infertility, IVF and the fertility clinic Access to own medical record with all available information concerning the patient’s IVF or ICSI treatment Tailored, context-sensitive clarification of clinical information Communication options such as e-mail, discussion forum, chat room	No access to the web site
Zhou, 2007 [24]	09/02 – 08/05	Kaiser Permanente Northwest Region, located in Oregon and Washington	Patients that used KP Health Connect Online for longer than 13 months and that had used at least one feature were invited.	Web-based access to KP HealthConnect, offering: Access of parts of their individual health record Health summary with problem list, medications, allergies Health record with immunizations Secure provider messaging Administrative requests (update medical record, appointments etc.) Visit-related inquiries such as after-visit summary, future appointments Educational materials	Period 3- 14 months before KP HealthConnect Online registration
Grant, 2008 [25]	09/05 – 03/07	11 primary care practices (with 230 physicians) within the Partners Health care System (Massachusetts)	Patients with diabetes mellitus type 2 who had at least one visit with their designated primary care provider in the study in the prior year, and who had logged in at least once in PatientGateway, the patient portal.	Access to a diabetes-mellitus-specific application offering: Medication module to review medications and edit inaccuracies View most recent results and current treatments (glucose, blood pressure, LDL-C, preventive care) Enter therapy concerns and request Answer short questions on therapy adherence and adverse effects Generate a diabetes care plan based on patients’ responses to the questions, to be used at the next clinical visit	Access to limited functionalities: Update family medical history; Review preventive services. Comment:Both groups (control and intervention) were active users of a general online portal called PatientGateway (PG), offering: Update registration information Confirm appointments Sending non-urgent clinical messages Request prescription refills
Earnest, 2004 [26] Ross, 2004 [27]	01/02 – 12/02	Academic subspecialty clinic for patients with congestive heart failure at University of Colorado Hospital, Denver, Colorado	Adult patients with congestive heart failure and internet access.	Access to web-interface of SPPARO (“System Providing Patients Access to Records Online”) offering: Online access to clinical notes, laboratory test results, other test results Patient information packet Send messages to the clinic and receive messages	No access to the SPPARO

**Table 2:** Details of included studies – study design and outcome

Author/ Year	Study description	Outcome criteria (differentiated between primary and secondary outcome) and finding of study (first number intervention group, second number control group)
Tuil, 2007 [23]	Randomized controlled trial Duration of intervention: unclear Number of participants in intervention group: 102 patients Number of participants in control group: 78 patients	Primary endpoint: Patient empowerment measured as multidimensional concept composed of: General Self-Efficacy scale and IVF-specific self-efficacy measure Objective knowledge about IVF treatment Subjective knowledge level regarding IVF treatment Problem-Solving Decision-Making Scale Further used instruments on secondary variables: Patient Satisfaction Questionnaire Illness Cognition Questionnaire Inventory for Social Support State-Trait Anxiety Inventory Beck Depression Index for Primary Care Utrecht Coping List Result: There were no statistically significant changes and no differences between effect measures on the above listed variables, measured by validated questionnaires in both groups pre and post: Summary by the authors: “The interactive online medical record did not result in significant changes in patient empowerment.”
Zhou, 2007 [24]	Retrospective matched-control study comparing 3-14 months before and 2-13 months after registration of the user in the portal The intervention group was compared to a control group matched by age, sex, selected chronic conditions and primary care physician. Duration of intervention: 2 – 13 months Number of participants in intervention group: 3,201 patients Number of participants in control group: 3,201 patients	Primary endpoint: Physician workload measured as primary care office visit, documented telephone contact rates Annual adult primary care office visit rates in the intervention group decreased from 2.44 (95% CI 2.35-3.54) to 2.19 (95% CI 2.11-2.27) (=10.3%) (*P*<0.001). Annual adult primary care office visit rates in the control group decreased from 2.15 (95% CI 2.08- 2.23) to 2.07 (95% CI 2.00-2.15) (=3.7%) (*P*<0.003). The difference in decrease -0.17 (*P*<0.01) (=6.7%) between both groups was statistically significant (p<0.003). Documented telephone contact rates in the intervention group increased from 2.0 (95% CI 1.89- 2.11) to 2.32 (95% CI 2.21-2.43) (=16.2%) (*P*<0.001).Documented telephone contact rates in the control group increased from 1. 74 (95% CI 1.63-1.85) to 2.26 (95% CI 2.14-2.37) (=29.9%) (*P*<0.001). The difference in increase 0.20 (*P*<0.001) (=13.7%) between both groups is statistically significant (*P*<0.01). Conclusion by the authors: “Patient access to the secure messaging feature of KP HealthConnect Online was associated with decreased rates of primary care office visits and a smaller increase in documented telephone contacts.”
Grant, 2008 [25]	Cluster randomized controlled trial Practices were grouped in 4 strata; practices within each stratum were then randomly assigned to either intervention or control arm. Duration of intervention: 12 months Number of participants in intervention group: 4 practices with 126 patients Number of participants in control group: 7 practices with 118 patients	Primary endpoint: More effective treatment of DM-related risk factors (hyperglycemia, hypertension, hyperlipidemia), measured by: Decline in HbA1c after one year: 0.16% vs. 0.26% (*P*=0.62) Mean HbA1c after 1 year 7,1% vs. 7,2% (*P*=0.45) Changes in blood pressure after one year: slight improvement, no significant differences between groups (data not shown) Changes in LDL-C after one year: slight improvement, no significant differences between groups (data not shown)Subgroup of patients who submitted PHR journals: Changes in DM-related medications in subsequent care episodes: 53% vs. 15% (*P*<0.001) Medication adjustment for hyperglycemia: 29% vs. 15% (*P*=0.1)Medication adjustment for hypertension: 13% vs. 0% (*P*=0.02) Medication adjustment for hyperlipidemia: 11% vs. 0% (*P*=0.03) Conclusion by the authors: “Users of the diabetes mellitus-specific PHR were markedly more likely to have their medical regimens changed at their next clinic visit. Lack of an overall impact on DM-related risk factor levels.”
Earnest, 2004 [26] Ross, 2004 [27]	Randomized controlled trial: Duration of intervention: 12 months Number of participants in intervention group: 38 patients Number of participants in control group: 43 patients	Primary endpoint: Change in the self-efficacy domain of the Kansas City Cardiomyopathy Questionnaire with minimal significant difference of 7.7 Health status (measured by Kansas City Cardiomyopathy Questionnaire scored from 0 to 100): Difference in change between intervention and control group after 12 months: Self-efficacy domain: +6 points (95% CI 1 - 11), *P*=0.08 Symptom stability domain: +17 points (95%-CI 4-29), *P*<0.01, *P*<0.06 adjusted for multiple comparisons Not statistically significant differences between groups after 12 months between -4 to +2 points in the subdomains “symptoms”, “quality of life”, “functional status”, “clinical summary”, “physical limitations”. (“a change of 5 points is considered clinically important”) Patient satisfaction (measured with the adapted Art of Medicine Questionnaire scored from 1 to 5): 6 subitems with differences between -0.2 to +0.4 points between groups, not statistically significant after adjustment for multiple comparisons. Adherence to medication (measured by Morisky questionnaire scored from 0 to 4)): Difference between intervention and control group after 12 months +0.2 p=0.15 General adherence to medical regimens (measured by General Adherence Scale scored from 0 to 100): Difference between intervention and control group after 12 months +6.4 p=0.02 adjusted Phone and electronic messages: Number of total messages per patient (phone + electronic) in the intervention group was significantly higher 350 vs. 267 (*P*=0.02) Mortality: 11% in intervention group, 11% in control group, p=1.0 Emergency department visits:Number of patients visiting an emergency room: 20% in intervention group, 13% in control group, *P*=0.44 Number of visits in an emergency room: 20 in intervention group, 8, in control group, *P* = 0.03 Hospitalizations: 20% in intervention group, 23% in control group, *P*=0.81 Heart failure practice visits: 93% in intervention group, 92% in control group, *P*=1.0 Patient Empowerment Score (self-defined, calculated from 8 questions with a 5-point Likert scale): Patient Empowerment Score both at baseline as well as after 12 months did not show significant differences between intervention and control group (data not shown in the study) Patient Empowerment Score in both groups declined between baseline and 12 months (from 89% to 74% that agreed with at least 4 of the 8 questions, *P*=0.01). Summary by the authors: “No differences developed between the subject and the control groups, but the Patient Empowerment Score declined for patients as a whole… We did not demonstrate a significant effect on self-efficacy, [but] there was an improvement in general adherence to medical advice, and there were trends towards improvement in patient satisfaction with doctor-patient communication.”

### Risk of Bias

Overall risk of bias is unclear in 3 studies [24-27] and high for 1 study [23]. One RCT had no adequate concealment of allocation; for two RCTs the information was lacking. In the observational study, groups were matched for primary care physician, age, sex, and selected chronic conditions, but were potentially confounded by other factors eg, education cannot be excluded. Whether this could have led to a bias is unclear.

In addition, in the 3 prospective studies, due to the nature of the intervention, patients and clinicians were not completely blinded to patients’ allocation. For example, as soon as the patient contacts the physician via secure messaging or brings along print-outs of the portal’s information, his or her allocation is known to the clinician. It is unclear, however, whether this may have led to a more intensive treatment of the patient and, if yes, whether this can be seen as a desired effect of portals, or as a possible source of bias. One study [23] additionally suffered from high drop-out rates. Here, too, the impact on the results is not clear.

The studies used different methods and instruments to assess different types of impact. In most cases, the used questionnaires were based on validated survey instruments. Table 3 summarizes the risk of bias of each study. For details on each criterion, please see the NICE checklists for the respective RCT cohort studies [22].

**Table 3:** Assessment of the quality of studies

Question			Grant [25]	Earnest [26] Ross [27]	Tuil [23]	Zhou [24]
**Type of study**			RCT	RCT	RCT	Matched- control study
			(checklist: RCT)	(checklist: RCT)	(checklist: RCT)	(checklist: cohort study)
**Selection bias (A1 – A3)**			unclear ^a^	unclear	unclear	unclear
	A1	*Appropriate method of randomization* was used to allocate participants to treatment groups (for RCT)	unclear	yes^b^	no^c^ (used order of reception of forms)	Not relevant
		*Method of allocation to treatment groups* was unrelated to potential confounding factors (for cohort study)	not relevant	not relevant	not relevant	unclear
	A2	*Adequate concealment of allocation*, such that investigators, clinicians and participants cannot influence enrolment or treatment allocation (for RCT)	unclear	unclear	no (uses alternate allocation)	not relevant
		Any attempts made to *balance* the comparison groups *for potential confounders* (for cohort study)	not relevant	not relevant	not relevant	yes
	A3	Groups were *comparable at baseline*, including all major confounders/prognostic factors	no (differences in age)	unclear	yes	yes
**Performance bias (B1 – B3)**			unclear	unclear	unclear	low risk
	B1	Comparison groups *received the same care* apart from the interventions	unclear	unclear	unclear	yes
	B2	*Patients* receiving intervention were *kept blind* to treatment allocation	unclear	no	no	yes
	B3	*Clinicians* were *kept blind* to treatment allocation	unclear	no	no	yes
**Attrition bias (C1 – C3)**			low risk	low risk	high risk	low risk
	C1	All groups were *followed up* for an equal length of time	yes	yes	unclear	no
	C2	Groups were *comparable for treatment completion*.	yes	yes	nodrop-outs in control groups	yes
	C3	Groups were comparable with respect of the *availability of outcome data*.	yes	yes	nodrop-outs in control groups	yes
**Detection bias (D1 – D5)**			unclear	low risk	unclear	unclear
	D1	Study had an *appropriate length of follow-up*	yes	yes	unclear	unclear
	D2	Study employed a *precise definition of outcome*	yes	yes	yes	yes
	D3	Study used a *valid and reliable method* to determine the outcome	unclear	yes	yes	unclear
	D4	*Investigators* were kept *blind* to patients’ exposure to the intervention	unclear	unclear	unclear	unclear
	D5	*Investigators* were kept *blind* to other important confounding/prognostic factors	unclear	unclear	unclear	unclear
**Overall rating**			unclear	unclear	high risk	unclear

^a^ unclear = not sufficient information in the paper to assess quality criterion

^b^ yes = criterion is fulfilled

^c^ no = criterion is not fulfilled

## Discussion

We systematically searched the literature and found 4 controlled studies focusing on the impact of electronic patient portals. Given the fact that patient portals have been in use in the U.S. for several years, the number of controlled studies seems quite low.

The studies were quite heterogeneous with regard to clinical setting, functionality of the intervention, and measured outcome. The different outcome parameters used made any further aggregation of results impossible, and showed how little evidence is available for each single outcome parameter. Most of the measured parameters did not show a statistically significant difference between intervention and control group. In particular, no statistically significant changes could be observed for parameters related to clinical outcome. Two studies found changes in contact patterns: quicker decrease in office visit rates and smaller increase in telephone contacts [24]; increase in number of messages sent [27]. Two studies found changes in medication regimen: higher changes of medication regimen [24]; better adherence to medication [27].

### Impact of Patient Portals

We defined patient portals as presenting clinical information to the patients. Can we expect that giving patients access to clinical information can in general have an impact? The review of Ross et al [8] - who is, also the author of one of the studies we reviewed [27] - was dedicated to this question. It reviewed the outcome of 29 descriptive or controlled studies on adult patients’ access to (paper-based) medical records. Several studies showed an improvement of doctor-patient communication by patient-accessible medical records. There were, however, conflicting findings on improvements in treatment adherence, patient education, and patient empowerment; some controlled studies showed an improvement, while others did not. Ross et al [8] summarized that studies show potential for modest benefits, for example in enhancing doctor-patient communication, but that more research is necessary.

Compared to paper-based access to records, electronic (web-based) patient portals allow a patient to access the information independently and repeatedly; the information is better legible; and the user can link the information to further sources of medical information available on the Internet [8]. Also, patient portals can be adapted to the patient’s wishes and knowledge level [23]. They can also be completed by secure communication links with health care providers or other functions. Overall, we could expect a higher impact of online portals compared to paper-based access. However, as our results show, the impact of patient portals, indicated by the studies reviewed here, is of a limited nature. In the following sections, we will discuss the findings of the 4 studies with regard to different topics.

### Impact on Clinical Outcome

Grant et al [25] assessed changes in clinical parameters related to diabetes patients (such as HbA1, blood pressure, and LDL-C). He did not find statistically significant differences between both groups in general. But he found statistically significantly higher rates for medication adjustments of diabetes-mellitus related drugs. The portal they evaluated, however, included a module where a diabetes care plan was generated automatically based on the patient’s responses to short questions; these care plans may have led to the higher rate of medication adjustments in the intervention group, not so much the presentation of clinical data itself. In the study of Ross et al [27], mortality was compared between both groups and no differences could be seen. Overall, there are not sufficient studies to decide on the impact of patient portals on clinical outcomes.

### Impact on Health Resource Consumption

Zhou et al [24] found a stronger decrease of annual primary care office visits in the intervention group compared to the control group when the intervention group used a patient portal with secure messaging. As explanation, Zhou mentions that a quarter of portal users indicated they would have scheduled an appointment in lieu of electronic messaging; so there seems to be a possibility of saving resources by a portal with electronic messaging. Ross et al [27] assessed a statistically significant increase of visits in the emergency room in the intervention group, but without temporal relationship between portal use and visits; also, no differences in hospitalization or visits to heart failure practice visits were observed. Thus, while the study of Zhou et al may indicate that a portal with electronic messaging may reduce the number of office visits, there is not sufficient data to decide conclusively on this.

### Impact on Patient Adherence

Only the study of Ross et al [27] assessed adherence and found an increase in general adherence; this was measured by a validated questionnaire, not by objective data. Adherence to medication also increased, but did not reach statistical significance. In an earlier review, Ross et al [8] found 1 study with increased adherence, but 5 studies that could not support this. It seems plausible that better-informed patients show higher adherence to treatment or to clinical advices, but there is not sufficient evidence to support this assumption.

### Impact on Patient-Physician Communication

Some of the reviewed studies addressed aspects of patient-physician communication. The general patient portal (KP HealthConnect) assessed by Zhou et al led to a slower increase of telephone contacts, and a quicker decrease of primary care office visits, compared to the control group [24]. In the study of Ross et al, the SPPARO portal for patients with congestive heart failure led to a statistically significant increase of the number of overall messages (electronic + phone) per patients [27], compared to the control group that just used phone. The authors argue here that SPPARO “appeared to supplement, rather than replace, telephone messages”. A consistent finding of these changes, or any related change in quality of communication, is not possible based on this data.

### Impact on Patient Empowerment

Three of the reviewed studies addressed the concept of patient empowerment. The term “patient empowerment” has been controversially discussed in the literature [28], and a generally accepted definition seems to be missing [23, 29]. Consequently, each of the found studies used a different approach to measure patient empowerment.

Tuil et al [23] measured a multidimensional concept composed of self-efficacy (using the General Self-Efficacy Scale [30]), actual and perceived knowledge, and patients’ involvement in the decision process. He did not find a statistically significant impact on any of those scales. Earnest et al [26] used a self-developed patient empowerment scale consisting of 8 questions (including feeling more in control, better prepared, feeling more reassured, trust, etc.); this study found a statistically significant decrease in patient empowerment scale scores over the study period in both groups, but no significant differences between both groups. Ross et al used the Kansas City Cardiomyopathy Questionnaire [31] to assess the health status of the participants; one subscale of it is devoted to self-efficacy. He found a trend (p=0.08) for an increase in self-efficacy in the intervention group, but with 6 points it was less than the predefined meaningful difference of 7.7 points. Overall, no study was able to show impact on patient empowerment. In their review of paper-based record access, Ross et al [8] also found some improvement in patient empowerment in randomized controlled trials, where patients felt “more in control” and “less helpless”; however, other controlled trials failed to support this finding. Overall, portals may have an impact on patient empowerment, but the evidence is not sufficient on this question.

A review conducted by Samoocha [29] about the effectiveness of web-based interventions on patient empowerment arrives at the same conclusion. There are disease-specific self-efficacy effects that could be found, but a general increase in self-efficacy could not be identified, as evidence is not sufficient.

### Impact on Patient Satisfaction

Tuil et al [23] used the Patient Satisfaction Questionnaire [32] and found no differences between both groups. Ross et al [27] assessed patient satisfaction with the Art of Medicine Questionnaire and found improvements in two questions: How well patients felt their problems were understood, and how well doctors explained information. For the other four questions, no impact was seen. After adjustment for multiple testing, there was no statistically significant effect at all. The review of paper-based access of Ross et al [8] found 6 studies focusing on this topic, none of them showed statistically significant differences. Overall, access to information is probably only one facet of patient satisfaction; it is therefore questionable whether the impact of a patient portal on patient satisfaction is measurable.

### Meaning and Generalizability of Findings

The results presented by the 4 studies did not contain convincing evidence for a general positive impact of electronic patient portals on clinical outcome, resource consumption, patient satisfaction or other variables compared to conventional ways of communication. Three of 4 studies were conducted in the U.S. The generalizability to health care settings in other countries is unclear.

Outcome research regarding patient portals is still at its beginning, and most of the analyzed studies could not show clear benefits for the patient regarding quality of patient care. Given the large resources needed to build and maintain patient portals, health care institutions should carefully weigh costs and (expected) benefits.

There may be several explanations for the missing evidence of the benefit of patient portals:

Electronic portals provide information from the medical record to patients. However, better-informed patients are not necessarily healthier patients [27, 33]. Descriptive evidence from a large number of studies suggests that patients are interested in access to their patient records, and that they find it helpful and useful [10, 34-36]. These findings, however, do not guarantee that there is in fact a measurable impact on health, as a better-informed patient is only one (possibly minor) factor contributing to the quality of care.

Studies in which a patient portal was combined with further services, such as secure messaging, interactive decision-support or health-related reminders, showed more positive impact on patient outcomes, patient-provider communication, disease management, and patient satisfaction, as a recent review of diabetes portals showed [37]. The interactive guiding and coaching of patients may be more effective than purely presenting clinical information without further advice.

Especially patients with chronic diseases (for example, with diabetes mellitus, congestive heart failure) and patients with intensive and long-time treatment (for example, IVF) may be more willing to use electronic portals [27]. Nevertheless, these groups may be already actively communicating with their physicians, therefore a portal does not show additional impact. This could explain that the studies in our review (with 3 of 4 studies focusing on these types of patients) did not show statistically significant impact.

Finally, only a minority of patients may be interested in using patient portals. Less computer literate, less motivated or less ill patients may not be interested. For example, in the Kaiser Permanente Northwest region (Oregon and southwest Washington), only 6% of all members have registered to the patient portal [24], and Weingart reports [38] a 11% utilization rate among primary care patients. Some study authors report difficulties in recruiting participants for the study [25], and some found that study participants are typically higher educated and have higher income than non-participants [27]. All this leads to the question whether patient portals may increase the digital divide, an issue also discussed by others [38].

### Limitations

#### Quality of the Studies

Three studies had sample sizes with less than 200 patients. Only one (retrospective) study included more than 6,000 patients. There was one study with a high risk of bias and no study with a low risk of bias. Crucial criteria to assess the risk of study bias, such as randomization method, concealment of allocation or blinding, were not reported in all publications.

None of the studies gave clear information as to how often the participants in the study group used the portal. Only the authors of the SPPARO study [26,27] mentioned that 80% of participants used the portal at least once, with a median of eight days during a one-year study period, which correlated roughly to the number of office visits during this time. The authors concluded that the patients did not use the portal repeatedly between these visits. In the other studies, no information on usage patterns is given; therefore, it is not clear whether the patients really exploited the offered functionalities. This may have reduced the measurable impact of the portals.

All electronic patient portals included in this review, offered functionality in addition to sole access clinical data. This renders comparison of studies difficult. In addition, it makes it difficult to identify to which functionality (clinical data access or additional functions) the measured effect can be attributed.

#### Completeness of the Review

We conducted a systematic literature search, but may have overlooked studies that were unpublished or in the grey literature.

With 4 identified studies, the available evidence is quite limited. Despite a comprehensive query, we cannot be sure to have identified all related studies, as the terms used in the title, abstract, and keywords are not uniformly used. For example, the concept “patient portal” has been circumscribed in the literature as shared medical record, access to medical record, online PHR, or online medical record.

It can be questioned how the benefit of patient portals can be operationalized at all – did the studies use the correct operators (e.g. number of phone contacts)? In the future, more research seems necessary on meaningful indicators that measure the effects of patient portals, and more patient portals should undergo systematic evaluation studies.

### Conclusion

Even if electronic patient portals are often seen as a way to empower patients and improve patient care, the available evidence does not support this assumption. Further studies of larger sample size and with harmonized outcome indicators are needed to investigate this question.

## Multimedia Appendix 1

Excluded papers.

## Abbreviations

DM: Diabetes Mellitus
EHR: Electronic Health Record
ICSI: Intracytoplasmic Sperm Injection
IVF: In-Vitro Fertilization
NICE: National Institute for Health and Clinical Excellence
PHR: Personal Health Record
RCT: Randomized Controlled Trial
SPPARO: System Providing Patients Access to Records Online
